# Récidive de mélanome malin unguéal achromique: à propos d'un cas

**DOI:** 10.11604/pamj.2015.22.320.8319

**Published:** 2015-12-02

**Authors:** Youssef Benyass, Bouchaib Chafry, Kaldadak Koufagued, Salim Bouabid, Driss Benchebba, Belkacem Chagar

**Affiliations:** 1 Service de Traumatologie-Orthopédie II, Hôpital Militaire d'Instruction Mohamed V, Rabat, Maroc

**Keywords:** Mélanome, récidive, chirurgie, Melanoma, recurrence, surgery

## Abstract

Le mélanome malin unguéal représente 1,8 à 8,1% des mélanomes malins cutanés. Sa prise en charge s'adresse aujourd'hui aux praticiens de différentes spécialités. L'acte chirurgical initial est une étape incontournable du traitement curatif. La biopsie de la lésion doit être complète, afin de déterminer de façon exacte la profondeur de l'envahissement en cas de malignité. Nous rapportons un cas de mélanome malin achromique à localisation unguéal chez une femme. La chirurgie initiale consistait en une amputation transphalangienne proximale. L’évolution après deux ans était marquée par une récidive avec extension vers le carpe. Ayant subie une reprise chirurgicale avec une exérèse large. Le traitement des récidives est palliatif et vise à apporter un confort de vie au patient. Le principe du traitement fait appel à l'exérèse chirurgicale des lésions. Des alternatives thérapeutiques sont à l’étude.

## Introduction

Le mélanome est une tumeur maligne à haut potentiel métastatique développée aux dépens des mélanocytes, cellules spécialisées dans la production des pigments mélaniques responsables de la couleur de la peau, des cheveux et des yeux. C'est la principale cause de décès par cancer cutané. Le mélanome unguéal est une forme rare du mélanome malin. Il représente 1,8 à 8,1% des mélanomes malins cutanés (MMC) [1]. Il est le plus fréquent chez le sujet de couleur noire (15 à 35%). Au niveau unguéal, il est achromique dans 15 à 65% [1]. Sa prise en charge s'adresse aujourd'hui aux praticiens de différentes spécialités: les dermatologues, les chirurgiens traumatologues et les chirurgiens plasticiens. La mauvaise prise en charge du mélanome unguéale évolue vers la récidive locale, qui est un marqueur fort d'agressivité. Les récidives locorégionales cutanées sont le premier site dans 59% des cas [2]. Le traitement des récidives est palliatif et vise à apporter un confort de vie au patient. Des alternatives thérapeutiques, comme la perfusion de membre isolé, sont à l’étude et semblent apporter une réponse efficace même si temporaire. Ce travail a pour objectif de mettre le point sur l'agressivité de cette tumeur, son potentiel de récidive et les possibilités thérapeutiques.

## Patient et observation

**Figure 1:**
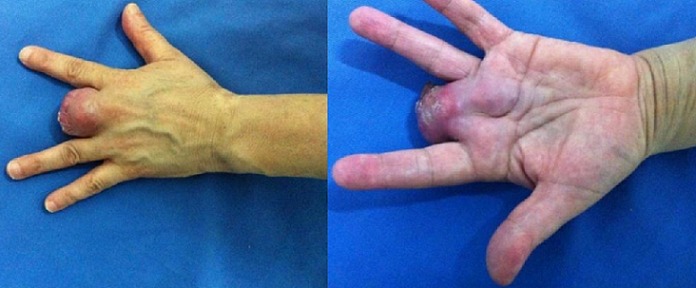
Aspect de récidive de mélanome après amputation

**Figure 2:**
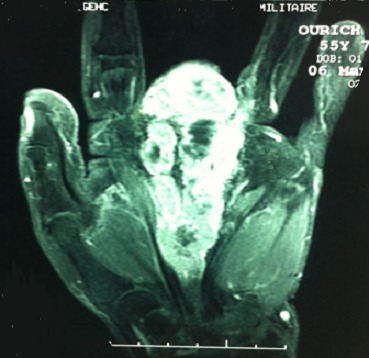
Imagerie par résonance magnétique montre l'extension tumorale

**Figure 3:**
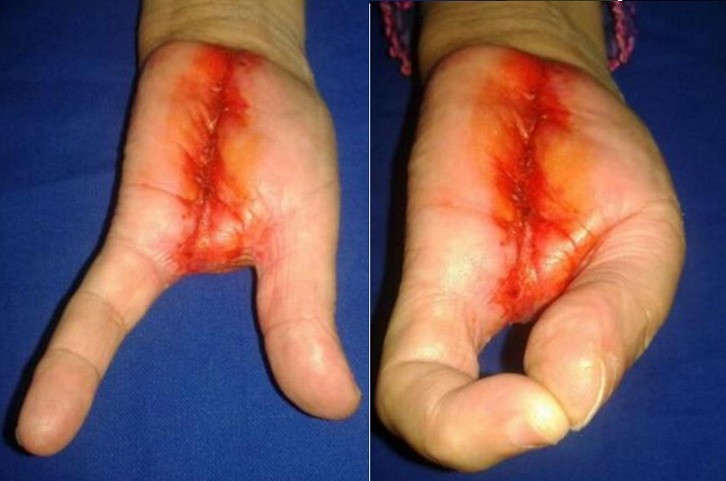
Résultat fonctionnel (pince type homard)

Il s'agissait de Mme H.O âgée de 55 ans connue diabétique type II sous traitement, qui a présenté une lésion ulcérée avec destruction partielle de la tablette unguéale du 3^ème^ doigt de la main gauche évoluant de façon progressive depuis 2 ans. L'installation d'une douleur au niveau du site a poussé la patiente a consulté dans notre formation. Après un bilan clinique et radiologique, une biopsie a été réalisée. L'analyse histologique a montré un mélanome malin acro-lentigineux ulcéré de niveau V de Clark et d'indice de Breslow de 4 mm. La patiente a subi une amputation transphalangienne proximale avec passage en zone saine. L'examen anatomopathologique de la pièce d'exérèse a confirmé la nature histologique de la lésion. Un bilan d'extension a été réalisé sans montrer de localisation secondaire. Aucun traitement complémentaire n'a été entrepris. Après 6mois, l’évolution a été marquée par l'absence de signe de récidive, puis la patiente n'a pas été revue. Un an après, la patiente a remarqué que le moignon de l'amputation devient inflammatoire avec augmentation de son volume. Elle a repris la consultation dans notre service. L'examen clinique a montré une tuméfaction du moignon qui a été rouge avec présence de nodule bleu dure et douloureux à la palpation, infiltrant la racine de l'index et l'annulaire (Figure 1). Par ailleurs, les aires ganglionnaires ont été libres. L'imagerie par résonnance magnétique a montré une extension atteignant le carpe (Figure 2). Le bilan d'extension à distance a objectivé une hyperfixation au niveau de la 8^ème^ cote à la scintigraphie. Le scanner thoraco-abdomino-pelvien a objectivé la présence de deux nodules pulmonaires. La Tomographie par émission de positons au 18-FDG a montré deux nodules pulmonaires contigus latéro basaux droits faiblement hyper métaboliques, d'allure secondaire et absence d'hyper métabolisme pathologique au niveau de l'ensemble du squelette exploré notamment au niveau du gril costal. La reprise chirurgicale a consisté en une amputation transverse courte intéressant 2^ème^, 3^ème^ et 4^ème^ rayons selon Chase et conservation complète de la colonne du pouce et de l'auriculaire tous en respectant des marges macroscopiques saines, et permettant à la patiente de garder une pince type celle de homard (Figure 3). Après avoir présenté le dossier aux oncologues, aucun traitement adjuvant n'a été entrepris. L'examen anatomopathologique de la pièce d'exérèse a montré une récidive de mélanome malin niveau V de Clark et indice de Breslow de 1,5 cm avec présence d'emboles vasculaires, d'engainements périnerveux et la confirmation des marges histologiques saines. La patiente a débuté la rééducation deux semaines après l'intervention pour la réadaptation rapide à la vie quotidienne. La patiente est revue régulièrement, son état clinique est stable, et elle est satisfaite de la fonction de sa main qui lui permet d'assurer ses taches quotidiennes.

## Discussion

Le mélanome a été mentionné par Hippocrate sous le terme de tumeur noire fatale, dès le Ve siècle avant Jésus Christ. Mais, c'est en 1838 que Carswell a utilisé pour la première fois le terme de mélanome pour désigner ces tumeurs mélanocytaires. Le mélanome de l'appareil unguéal est une variante anatomique du mélanome acro-lentigineux dont la première description détaillée a été attribuée à Hutchinson en 1886 (melanotic whitlow ou panaris mélanique) [3]. Le mélanome est une tumeur qui correspond à une excroissance pathologique de cellules aboutissant à un tissu néoformé retrouvé dans l'organisme, soit à l’état embryonnaire, soit à l’état adulte. IL peut être bénin, on parle alors de naevus, ou malin et on parlera alors de mélanoblastome. Mais, dans le langage médical, le terme de mélanome est réservé aux tumeurs malignes. Le mélanome malin cutané est en augmentation constante. En France, entre 1980 et 2000, l'incidence a augmenté de 2,4 à 7,6 pour 100 000 habitants par an pour les hommes, et de 3,9 à 9,5 pour 100 000 habitants par an pour les femmes [4]. L'incidence des mélanomes achromiques dans la littérature varie de 1,8 à 8,1% [1]. Il n'y a pas d'atteinte prédominante chez l'homme ou la femme [5]. Il survient chez le sujet âgé [2]. Le mélanome malin peut être achromique quelle que soit sa localisation. Malgré l'absence de pigmentation, il persiste des signes évocateurs de malignité (augmentation en taille de la lésion qui est mal limitée et asymétrique [5]). L'absence de mélanine peut se voir à la fois dans les mélanomes primitifs, les récidives locales et les métastases à distance. L'origine de l'achromie n'est pas connue précisément. Le mélanome achromique apparaît préférentiellement sur des zones photoexposées, mais cela n'a pas de rôle évident pour les localisations unguéales grâce à la tablette unguéale qui semble être un écran solaire efficace [6]. Il se retrouve plus fréquemment au niveau des extrémités, et plus particulièrement sous l'ongle [7]. Les mélanomes de l'appareil unguéal sont plus fréquents sur les doigts que sur les orteils [8]. La fréquente survenue sur le pouce et le gros orteil pourrait simplement être le fait d'une matrice unguéale beaucoup plus grande [8]. Dans la littérature, des auteurs avaient suggéré un lien possible entre le traumatisme et le mélanome du fait de la forte prépondérance des mélanomes de l'ongle sur le pouce et le gros orteil, qui sont plus exposés aux traumatismes [9]. Mais, le rôle des traumatismes sur la survenue des mélanomes n'a jamais été démontré sur aucun site. L’âge moyen du diagnostic du mélanome achromique est alors de 47 à 62 ans [10]. Ce plus long délai au diagnostic semble due à plusieurs facteurs: à une topographie négligée ou hors de portée de vue chez des personnes souvent âgées, à une présentation clinique trompeuse et à la fréquente achromie. Le taux d'erreur de diagnostic peut atteindre dans certaines études 12 à 68% [10]. Cliniquement, le mélanome malin unguéal se présente comme une bande longitudinale pigmentée et sombre unguéale (bande mélanique), associée plus au moins à une pigmentation du repli unguéal latéral ou proximal (signe de Hutchinson). Il peut prendre l'aspect d'une macule érythémateuse à bords irréguliers. La dermoscopie est une technique non invasive qui a permis une meilleure reconnaissance de ce sous-type rare de mélanome par les cliniciens. Elle est utile pour détecter des reliquats de pigmentation non visibles à l’œil nu. Les structures vasculaires sont souvent les seuls indices diagnostiques de mélanomes achromiques (≥ 3 patrons vasculaires associés au sien d'une même lésion) [11]. Les vaisseaux linéaires irréguliers, les zones rouges laiteuses, les vaisseaux en points, et le polymorphisme vasculaire sont le plus souvent associées au mélanome achromique. On peut rencontrer également un patron polymorphique et atypique avec des vaisseaux linéaires irréguliers, des vaisseaux en épingles à cheveux et des vaisseaux en points.

Devant une lésion pigmentée unguéale, l'attitude préconisée est la biopsie exérèse de l'intégralité de la lésion. Elle doit être complète et de pleine épaisseur afin de pouvoir déterminer de façon fiable la nature histologique de la lésion, et en cas de malignité, la profondeur de l'envahissement en termes d'indice de Breslow, et de niveau de Clark. On réalise une résection elliptique comprenant l'ongle (onycectomie partielle) jusqu'au périoste. La résection doit inclure la matrice jusqu’à sa partie la plus proximale à 5 mm du repli unguéal proximal (la réalisation d'un lambeau de rotation cutané est parfois nécessaire). D'autres préconisent une simple biopsie du lit de l'ongle (l'ongle est soulevé et remis en place ensuite) avec une marge de tissu sain macroscopique [12]. Une fois le diagnostic histologique est confirmé, il fait appelle à l'exérèse carcinologique, adapté à l’épaisseur tumorale mesurée selon l'indice de Breslow. Les marges d'exérèse de sécurité ont pour but théorique d’éliminer d’éventuelles micrométastases locales et de diminuer le risque de récidive locale et à distance. Actuellement, il est recommandé de se limiter à une amputation digitale à hauteur de l'interphalangienne distale pour les doigts longs et de l'interphalangienne pour le pouce [13]. Le système lymphatique est le principal mode de dissémination à distance du MMC. Le ganglion sentinelle (GS) correspond au premier relai de drainage lymphatique du MMC. Sa recherche a un intérêt pronostique qui est reconnu mais son intérêt thérapeutique est en cours d’évaluation. Les indications de cette procédure ont été clairement définit dans les recommandations de l'American Joint Commitee on Cancer (AJCC) [14]: indice de Breslow supérieur à 1 mm et inférieur à 4 mm; ulcération ou signes de régression clinique; absence d'adénopathie suspecte cliniquement (N0); Âge supérieur à 18 ans. L'identification du GS nécessite l'injection au site primitif du mélanome d'un traceur lymphophile selon la méthode colorimétrique par l'utilisant d'un colorant bleu, ou la méthode lymphoscintigraphique par l'utilisant des particules radioactives (radiocolloïde marqué). Cette procédure est réalisée en premier avant l'exérèse élargie de la cicatrice afin de ne pas sectionner les canaux lymphatiques efférents et risquer de compromettre la détection du GS. La récidive locale du mélanome malin est un marqueur fort d'agressivité. Dans la littérature, le mélanome achromique de l'appareil unguéal représente de mauvais pronostic et récidive plus vite que ceux ayant un mélanome pigmenté [15]. Cliniquement, elle se manifeste par la réapparition précoce ou tardive d'une tache noirâtre ou d'un nodule bleuté dans la cicatrice d'exérèse de la lésion primitive ou dans la zone reconstruite par plastie, par greffe ou par lambeau [7]. La récidive locale est dite vraie lorsqu’à l'analyse anatomopathologique on retrouve un contingent « in situ » associé. Le traitement chirurgical doit alors être celui d'une lésion primitive. Il consiste en une excision avec une marge macroscopique de tissu sain de 2 cm [16]. La récidive locorégionale englobe les récidives ganglionnaires régionales et les récidives cutanées en amont des aires ganglionnaires de drainage. Les récidives ganglionnaires sont traitées de la même façon que l'envahissement ganglionnaire primitif (curage). Les récidives locorégionales cutanées sont le premier site de récidive dans 59% des cas [2]. Elles englobent les lésions satellites et les métastases en transit. La survie est identique entre la présence de satellites et les métastases en transit ou les métastases ganglionnaires, la distance n'influant pas sur la survie [2]. La survenue de ces types de lésions est un facteur prédictif fort de métastases viscérales. Les sites métastatiques préférentiels sont, par ordre de fréquence décroissante: le poumon, le foie, le cerveau, et les os. La médiane de survie en cas de métastase à distance est de 6 à 8 mois [17].

Le traitement des récidives est palliatif et vise à apporter un confort de vie au patient. Le principe du traitement fait appel à l'exérèse chirurgicale des lésions. Cette exérèse est réalisée avec des marges macroscopiques saines. Certains auteurs Essner et al. recommandent une marge saine de 1 cm à laquelle ils rapportent une survie médiane de deux ans [18]. Le fort risque de propagation locale lors de la manipulation des métastases en transit implique une technique chirurgicale rigoureuse et protocolaire. En effet, la fracture d'un nodule en transit dans le champ opératoire peut être responsable d'une dissémination locale rendant le geste inefficace voire aggravant. L'utilisation d'un jeu d'instruments et de gants différents pour l'exérèse et la fermeture est recommandée [19]. Dans notre cas, la chirurgie réalisée chez notre patiente était une amputation transmétacarpienne intéressant les 2^ème^, 3^ème^ et 4^ème^ rayons selon Chase, ce qui a permis de garder le pouce et le 5éme rayon, et par conséquent assurer une opposition du premier rayon à la pulpe du 5^ème^ doigt permettant ainsi une pince type homard élément fonctionnel essentiel. Des alternatives thérapeutiques sont à l’étude et semblent apporter une réponse efficace même si temporaire. Comme la perfusion isolée de membre sous circulation extracorporelle par du Melphalan. Selon les études, cette technique permet d'obtenir un taux de 40 à 80% de réponse complète (disparition de toutes les métastases en transit) [20].

## Conclusion

Le mélanome malin peut être achromique quelle que soit sa localisation. Mais, il se retrouve plus fréquemment au niveau des extrémités et plus particulièrement sous l'ongle. L'examen clinique d'une lésion achromique doit donc être rigoureux avec la recherche d'une modification de sa taille, l'un des meilleurs critères en faveur d'un mélanome. La biopsie-exérèse a fait la preuve de son efficacité face aux autres techniques dans le diagnostic du MMC. L'acte chirurgical initial est une étape incontournable du traitement curatif. Les récidives locales et locorégionales répondent le plus souvent à la chirurgie d'exérèse qui doit être pratiquée avec des marges macroscopiques saines, bien que les procédures radicales d'amputation soient à éviter même en cas de lésions multiples. Des alternatives thérapeutiques sont à l’étude et semblent apporter une réponse efficace même si temporaire.
